# Epidemiology of vaccine-preventable diseases in Japan: considerations for pre-travel advice for the 2019 Rugby World Cup and 2020 Summer Olympic and Paralympic Games

**DOI:** 10.5365/wpsar.2017.8.4.002

**Published:** 2018-06-30

**Authors:** Matthew M. Griffith, Munehisa Fukusumi, Yusuke Kobayashi, Yusuke Matsui, Shingo Nishiki, Reiko Shimbashi, Saeko Morino, Tomimasa Sunagawa, Keiko Tanaka-Taya, Tamano Matsui, Kazunori Oishi

**Affiliations:** a Infectious Disease Surveillance Center, National Institute of Infectious Diseases, Tokyo, Japan.; b Field Epidemiology Training Program, National Institute of Infectious Diseases, Tokyo, Japan.

## Abstract

**Introduction:**

In 2019 and 2020, Japan will host two international sporting events estimated to draw a combined 22 million visitors. Mass gatherings like these ones increase the risk of spread of infectious disease outbreaks and international transmission. Pre-travel advice reduces that risk.

**Methods:**

To assist ministries of health and related organizations in developing pre-travel advice, we summarized national surveillance data in Japan (2000–2016, to the extent available) for rubella, invasive pneumococcal disease, measles, non-A and non-E viral hepatitis, hepatitis A, invasive *Haemophilus influenzae* disease, tetanus, typhoid fever, invasive meningococcal disease, Japanese encephalitis, influenza, varicella, mumps and pertussis by calculating descriptive statistics of reported cases and reviewing trends. (See **Annex A** for details of reviewed diseases.)

**Results:**

Our findings showed notable incidences of rubella (1.78 per 100 000 person-years), influenza (243.5 cases per sentinel site), and mumps (40.1 per sentinel site); seasonal increases for influenza (November–May) and Japanese encephalitis (August–November); and a geographical concentration of Japanese encephalitis in western Japan. Measles cases decreased from 11 013 in 2008 to 35 in 2015, but outbreaks (*n* = 165 cases) associated with importation occurred in 2016. Though invasive meningococcal disease incidence was only 0.03 per 100 000, international transmission occurred at a mass gathering in Japan in 2015.

**Discussion:**

Ministries of health and related organizations should use these findings to develop targeted pre-travel advice for travellers to the 2019 Rugby World Cup and the 2020 Summer Olympic and Paralympic Games, especially for mumps, measles, rubella, influenza, and meningitis. Travellers with increased exposure risk should also be advised about hepatitis A and Japanese encephalitis.

## Introduction

The 2019 Rugby World Cup will occur from 20 September to 2 November throughout Japan, and the 2020 Summer Olympic and Paralympic Games will happen in Tokyo from 24 July to 6 September. These mass gatherings (MGs) are estimated to attract 22 million visitors to Japan. (*1*) MGs like these can strain resources of the host country and have been associated with disease outbreaks and the international spread of disease. (*2*-*4*)

The World Health Organization (WHO) and Centers for Disease Control and Prevention (CDC) recommend travellers seek advice from health professionals before travelling to an MG. (*5*, *6*) This strategy has been associated with a twofold increase in vaccinations among Hajj pilgrims who seek such advice compared to those who do not. (*7*)

Up-to-date vaccination for all vaccine-preventable diseases (VPDs) is the best way to prevent illness, outbreaks and the international spread of disease. To assist ministries of health and other organizations in developing targeted pre-travel advice for these MGs, we aimed to summarize the recent epidemiology of selected VPDs in Japan.

## Methods

We selected diseases based on frequency, severity and potential immunity (i.e. likelihood that foreign travellers to Japan would have developed immunity against the disease before visiting Japan because of wide circulation of the pathogen or global vaccination trends) among visitors to Japan. We obtained data from the National Epidemiological Surveillance of Infectious Diseases (NESID) system for a period of at least eight years up to the latest finalized data (for most diseases 2015; years inclusive unless otherwise noted). National notifiable disease surveillance comprises passive case-based reporting from all health-care facilities in Japan. For this work, we selected rubella, invasive pneumococcal disease, measles, viral hepatitis non-E and non-A, hepatitis A, invasive *Haemophilus influenzae* disease, tetanus, typhoid, invasive meningococcal disease and Japanese encephalitis. NESID also has weekly sentinel surveillance from approximately 3000 paediatric clinics for some diseases. Of these, we selected varicella, mumps, pertussis and influenza. An additional 2000 adult outpatient clinics report influenza.

We gathered data on case totals by sex, age group, prefecture, and week and year of report. When available, we obtained clinical disease classifications (e.g. modified measles), vaccination history, the suspected location or route of infection and laboratory results. For influenza and varicella, we obtained counts of hospitalized cases with laboratory evidence of infection. Influenza cases have been reported from hospitals with more than 300 beds since 2011 and varicella cases from all hospitals since September 2014.

We calculated totals, proportions, ranges, and incidence per 100 000 person-years using annual population estimates from Japan’s Statistics Bureau, (*8*) applying relevant proportions for incomplete years. For sentinel diseases, we calculated mean cases reported per sentinel site because catchment population sizes were unavailable. To further contextualize the findings, we briefly described current vaccination policies. For detailed case definitions and additional disease information, **see Annex B**.

## Results

### VPDs under national notifiable disease surveillance (non-sentinel)

#### Rubella (2008–2015)

For the period reviewed, approximately 79% of the reported rubella cases occurred in 2013 (*n* = 14 344). The 2013 epidemic affected all 47 prefectures and comprised mostly males (76%; *n* = 10 972) and persons aged 20–44 years (70%; *n* = 10 055). Cases for the overall period were also mostly male (75%; *n* = 13 660) and aged 20–44 years (69%; *n* = 12 440). For 2015, cases with known vaccination history (*n* = 74; 45%) were typically undervaccinated: 49% (*n* = 36) unvaccinated, including four individuals too young to vaccinate, and 41% (*n* = 30) with one dose. The incidence of rubella for the reviewed period was 1.78 cases per 100 000 person-years (*n* = 18 117) (see **Table 1**).

**Table 1:** Incidence and characteristics of selected notifiable (non-sentinel) vaccine-preventable diseases (VPDs), Japan, 2006–2016

Disease (*n* cases; period reviewed)	Period incidence^a^	Annual incidence range^b^	% Male (*n*)	Predominant age groups in years:% (*n*)	Case reporting	Vaccination schedule^f^
**Rubella (18 117;** **2008–2015)**	**1.78**	**0.07–11.3**	**75 (13 660)**	**20–44: 69 (12 440)**	**Clinical or laboratory**	**Routine**
**IPD (5 229;** **2013^c^–2015)**	**1.50**	**1.05–1.9**	**60 (3 134)**	**< 5: 19 (1 017);** **≥** 60: 62 (3 227)	**Clinical and laboratory**	**Routine** **(PCV7 and PPSV23)**
**Measles (13 805;** **2008–2016)**	**1.20**	**0.03–8.6**	**57 (7 812)**	**20–39: 58 (95)^d^**	**Clinical or laboratory**	**Routine**
**Viral hepatitis^e^** **(2 454; 2006–2015)**	**0.19**	**0.17–0.22**	**74 (1 808)**	**20–44: 64 (1 559)**	**Clinical and laboratory**	**Routine** **(hepatitis B)**
**Hepatitis A (2 245;** **2006–2015)**	**0.18**	**0.10–0.34**	**58 (1 313)**	**25–64: 72 (1 744)**	**Laboratory**	**Voluntary**
**IHD (560;** **2013^c^–2015)**	**0.16**	**0.11–0.20**	**60 (338)**	**≥** 70: 52 (293)	**Clinical and laboratory**	**Routine**
**Tetanus (1 158;** **2006–2015)**	**0.09**	**0.07–0.10**	**56 (653)**	**≥** 55: 85 (984)	**Clinical**	**Routine**
**Typhoid (330;** **2008–2015)**	**0.03**	**0.02–0.05**	**59 (195)**	**20–39: 59 (194)**	**Laboratory**	**Not approved**
**IMD (94;** **2013–2015)**	**0.03**	**0.02–0.03**	**64 (60)**	**≥** 50: 59 (55)	**Clinical and laboratory**	**Voluntary (MCV4)**
**Japanese encephalitis (51; 2006–2015)**	**0.004**	**0.002–0.008**	**63 (32)**	**≥** 60: 61 (31)	**Clinical and laboratory**	**Routine**

a. Per 100 000 person-years; b. More than 4 consecutive weeks higher than the weekly average of reported cases for the year; c. From April; d. For 2016 (***n*** = 165); e. Non-A, non-E: 81% HBV; f. **See** immunization schedule in Japan (1 October 2016) at https://www.niid.go.jp/niid/images/vaccine/schedule/2016/EN20161001.pdf.

IHD: invasive *Haemophilus influenzae* disease

IMD: invasive meningococcal disease

IPD: invasive pneumococcal disease

MCV4: Meningococcal Conjugate Vaccine (Quadravalent)

PCV7: Pneumococcal Conjugate Vaccine (7-valent)

PPSV23: Pneumococcal Polysaccharide Vaccine (23-valent)

Since April 2006, the routine vaccination schedule has included two doses of measles-rubella (MR) vaccine: the first dose at 1 year of age and the second dose less than 1 year before entering primary school (typically age 5 or 6). Prior to 2006, a single rubella dose had been routine for 11–12-year-old girls since 1977. Doses for both sexes aged 12–89 months and for boys 11–12 years old were added in 1995.

#### Invasive pneumococcal disease (IPD) (2013–2015)

Reported cases of IPD were consistently lowest from mid-July to late September. The incidence was 1.5 per 100 000 person-years (*n* = 5229) from April 2013, when IPD became notifiable, through 2015. Adults aged ≥ 60 years accounted for 62% (*n* = 3227) of cases, while children < 5 years made up 19% (*n* = 1017). Males accounted for 60% (*n* = 3134).

Since November 2013, routine vaccination has included four doses of 13-valent pneumococcal conjugate vaccine (PCV13) for children aged 2–59 months. PCV13 replaced PCV7, which had been subsidized since November 2010 and was routine since April 2013 for children < 24 months old. Routine immunization with 23-valent pneumococcal polysaccharide vaccine for adults aged ≥ 65 years began in October 2014 after having been available on a voluntary basis since 1988.

#### Measles (2008–2016)

Reports of measles in Japan decreased from 11 013 cases in 2008 to 35 cases in 2015. In 2016, however, 165 cases were reported. Of these, 32% (*n* = 52) were *modified measles* (i.e. laboratory confirmation with less than 3 classic measles symptoms), 58% (*n* = 95) were 20–39 years old and 19% (*n* = 32) were < 10 years old. Among the 112 cases in 2016 (68%) with known vaccination history, most were undervaccinated: 42% (*n* = 47) unvaccinated, including eight under vaccine age and 36% (*n* = 40) with one dose. Measles virus was detected in 139 (84%) of the 2016 cases, 25% (*n* = 35) of which had travelled abroad. Genotype was identified for 89% (*n* = 124) of these isolates: 53% (*n* = 66) D8, 46% (*n* = 57) H1 and < 1% (*n* = 1) B3. Case-patients with these isolates who had not travelled abroad were linked to international airports in Japan. For the reviewed period, measles incidence was 1.2 per 100 000 person-years (*n* = 13 805).

See rubella vaccination (above). Additionally, a single measles vaccine dose has been available for children aged 12–71 months since 1978 and was expanded to 89 months in 1995.

#### Viral hepatitis (non-A, non-E) (2006–2015)

Hepatitis B virus (HBV) accounted for 81% (*n* = 1933) of the 2400 (incidence: 0.19 per 100 000 person-years) reported laboratory-confirmed viral hepatitis non-A, non-E cases in Japan for the reviewed period. HBV cases were 78% male (*n* = 1503). Suspected sexual transmission accounted for 70% of HBV cases (*n* = 1349). Of the 1091 male case-patients reporting sexual transmission, 66% (*n* = 715) reported heterosexual contact, 21% (*n* = 226) reported homosexual contact, 2% (*n* = 20) reported heterosexual and homosexual contact and 16% (*n* = 170) gave no response.

The routine schedule has included three doses of hepatitis B vaccine for infants aged < 12 months since October 2016. Voluntary maternal vaccination is also available.

#### Hepatitis A (2006–2016)

Hepatitis A cases peaked in 2006 (*n* = 320), 2010 (*n* = 347) and 2014 (*n* = 433) with higher frequencies during the first half of each year. Males were 58% of all cases (*n* = 1313) and persons aged 25–64 years were 72% (*n* = 1744). Domestic infection was suspected for 80% (*n* = 1185) of cases from 2010 to 2015 (prior data not reviewed) without regional clustering. Total incidence for the reviewed period was 0.18 per 100 000 person-years (*n* = 2245).

Since March 2013, two-dose inactivated hepatitis A vaccination has been available on a voluntary basis for all ages. Previously it had been available for those aged ≥ 16 years.

#### *Invasive* *Haemophilus influenzae* *disease (IHD) (2013–2015)*

The number of reported IHD cases increased from 108 in 2013 (IHD became notifiable in April 2013) to 200 in 2014 and 252 in 2015. Males accounted for 60% (*n* = 338) of the cases. Over half (52%; *n* = 293) of the cases were aged ≥ 70 years and 17% (*n* = 95) were < 5 years old. The incidence of IHD was 0.16 per 100 000 person-years (*n* = 560) from April 2013 through 2015.

Since April 2013, four doses of *H. influenzae* type b vaccine have been routine for those aged < 59 months. Voluntary vaccination for children < 5 years had been approved since December 2008; government financial assistance was added in November 2010.

#### Tetanus (2006–2015)

Between 89 and 128 cases of tetanus were reported each year with consistent increases during epidemiologic weeks 19–29. Cases were mostly aged ≥ 55 years (85%; *n* = 984). All prefectures reported cases. The incidence of tetanus in Japan for the reviewed period was 0.09 per 100 000 person-years (*n* = 1158).

Four doses of diptheria, tetanus, acellular pertussis, and inactivated polio vaccine (DTaP–IPV) between age 3 months and 7½ years and one diptheria and tetanus (DT) dose at age 11 or 12 are included in the routine schedule.

#### Typhoid fever (2008–2015)

Most cases of typhoid fever reported in Japan (72%; *n* = 238) were acquired outside Japan. The annual percentage of domestically acquired cases decreased from 38% (*n* = 25) in 2013 to 11% (*n* = 4) in 2015. Domestic cases appeared mostly sporadically with no known cause; however, in August 2014, an outbreak of eight cases was linked to salad consumption at a restaurant in Tokyo. (*9*) The incidence of typhoid fever for the reviewed period was 0.03 per 100 000 person-years (*n* = 330).

No vaccine has been approved for typhoid fever in Japan. Individual physicians may import and administer the vaccine without government reimbursement or, in the case of adverse events, patient compensation.

#### Invasive meningococcal disease (IMD) (2013–2015)

There were 23–37 cases of IMD reported each year from April 2013 (when meningococcal sepsis was added to the list of conditions requiring mandatory reporting for IMD) through 2015 with no seasonality. Most cases were male (64%; *n* = 60) and aged ≥ 50 years (59%; *n* = 55). IMD incidence in Japan was 0.03 per 100 000 (*n* = 94).

The meningococcal conjugate vaccine (MCV4) became available for voluntary use in May 2015.

#### Japanese encephalitis (JE) (2006–2015)

Of the 47 prefectures in Japan, five in western Japan (Fukuoka, Kumamoto, Nagasaki, Shimane and Ehime) accounted for 43% (*n* = 22) of reported JE cases for the reviewed period; 22 prefectures did not report any cases. Reports were consistently higher during epidemiologic weeks 35–47. Males were 63% (*n* = 32) and persons aged ≥ 60 years were 61% (*n* = 31) of all cases. JE incidence in Japan was 0.004 per 100 000 person-years (*n* = 51) with 2–10 cases reported each year.

Four doses of inactivated JE vaccine are included in the routine schedule: three between 6 months and 7½ years of age and one between 9 and 13 years of age.

### VPDs under sentinel surveillance

#### Influenza (2000–2015)

All influenza seasons reviewed, except 2009, began in November, peaked in late January to mid-March and finished in May. Sentinel sites reported 18 508 470 cases, averaging 243.5 annual cases per sentinel site (see **Table 2**). There were 9905 (2013–2014 season) and 12 705 (2014–2015 season) hospitalized cases with laboratory evidence of infection. No human infection with avian influenza A(H5N1), A(H5N6), A(H7N9) or A(H9N2) has been reported in Japan.

**Table 2:** Comparison of case and case-per-site totals and characteristics of reported selected sentinel based VPDs, Japan, 2000–2015

Disease (period reviewed)	Total cases	Cases per site (yearly range)	Range of *n* sites per year	Year-to-year trend	High season	Case reporting	Vaccination
**Influenza** **(2000–2015)**	**18 508 470**	**243.5** **(56.4–643.3)**	**4477–4924**	**Peaks every 2–3 years**	**Nov–May; peak: Jan–Mar**	**Clinical or rapid-kit detection of influenza A or B**	Voluntary; routine for some*
**Varicella** **(2006–2015)**	**2 018 171**	**65.5** **(24.7–88.1)**	**3012–3146**	**Overall decrease**	**Nov–June early on then none**	**Clinical**	**Routine**
**Mumps** **(2000–2015)**	**1 936 679**	**40.1** **(13.1–84.4)**	**2978–3146**	**Peaks every 4 years**	**None**	**Clinical**	**Voluntary**
**Pertussis** **(2000–2015)**	**48 783**	**1.0** **(0.44–2.24)**	**2978–3146**	**Fluctuating**	**None**	**Clinical**	**Routine**

* Those aged > 65 and those 60–65 with certain chronic diseases or immunocompromised conditions.

Seasonal influenza vaccinations for those aged > 64 years or 60–64 years with certain chronic diseases or immunocompromised conditions are in the routine schedule. For anyone else, vaccinations are voluntary.

#### Varicella (2005–2016)

A peak of 88.1 varicella cases per sentinel site (*n* = 265 453) was reported in 2006. The ratio decreased to 67.1 (*n* = 202 732) in 2009, increased to 76.2 (*n* = 238 645) in 2011 and decreased to 24.7 (*n* = 77 614) in 2015. Early in the reviewed period, cases peaked in November–June, but later they did not. Children < 5 years old represented 77% of cases in 2005–2011 and 54% in 2015. In total, 2 018 171 cases were reported from sentinel sites for 2005–2016 (65.5 cases per site per year). There were 521 hospitalized cases (clinically diagnosed or with laboratory evidence of infection) reported in Japan from mid-September 2014 through March 2016 (0.27 per 100 000 person-years).

Since October 2014, the routine vaccination schedule has included two varicella vaccination doses for children between 1 and 2 years old. The vaccine is available on a voluntary basis for those aged ≥ 2 years.

#### Mumps (2000–2015)

Mumps cases in Japan peaked in 2001 (84.4 cases per sentinel site), 2006 (66.6 cases per site) and 2010 (59.3 per site) without seasonality. No prefecture consistently reported high numbers of cases. Cases aged 2–5 years accounted for 57% (*n* = 1 048 851) and males for 54% (*n* = 1 051 903) of cases. In total, 1 963 679 cases were reported from sentinel sites from 2000 to 2015 (40.1 per sentinel site per year).

A monovalent mumps vaccination replaced the measles, mumps and rubella (MMR) vaccine in 1993 and is available on a voluntary basis for those aged at least 1 year.

#### Pertussis (2000–2015)

The number of reported pertussis cases per sentinel site fluctuated during the reviewed period: 1.28 (*n* = 3804) in 2000, 0.44 (*n* = 1358) in 2005, 2.24 (*n* = 6753) in 2008, 0.53 (*n* = 1662) in 2013 and 0.85 (*n* = 2675) in 2015. We did not observe seasonality. In 2001, 27% (*n* = 471) of the cases were 6–11 months old and 3% (*n* = 49) were aged ≥ 20 years. By 2010, those aged ≥ 20 years were 48% (*n* = 2607) and those 6–11 months were 4% (*n* = 205) of cases. For the 2000–2015 period, 48 783 pertussis cases were reported from sentinel sites (0.996 per sentinel site per year).

Four doses of DTaP-IPV are included in the routine schedule between ages 3 months and 7½ years.

## Discussion

Most VPDs in Japan present low risk for the majority of travellers attending the 2019 Rugby World Cup and 2020 Tokyo Summer Olympic and Paralympic Games. Occurrence has either declined or maintained a low level. Rubella, mumps, influenza, measles and IMD, however, present more complicated pictures. Hepatitis A and JE may pose higher risk for some travellers as discussed below.

Due to the epidemiology of rubella, mumps and influenza in Japan, these diseases should be prioritized for pre-travel advice. Rubella surged in 2013, likely related to undervaccination among adult males. A rubella antibody seroprevalence study in Japan in 2016 suggested that males 35–54 years old had less immunity than women for that age group; the gap narrowed to < 10 percentage points for those aged 20–34 and ≥ 55. (*10*) The vaccine was introduced in 1977 for 11–12-year old girls and was expanded in 1995 to boys 11–12-year-old and both sexes 12–89 months old. (*11*)

For mumps, 4–5 year peak cycles are also likely related to undervaccination. Recent mumps vaccination coverage in Japan has been 30–40%. (*12*) Vaccinations against mumps were voluntary until 1989 when MMR became routine; due to concerns with mumps component-related aseptic meningitis, MMR was replaced with a voluntary monovalent mumps vaccination in 1993. Mumps outbreaks with up to 214 cases have been reported at MGs in Europe. (*13*, *14*)

For influenza, seasons typically occur outside of when MGs are scheduled to occur in Japan. Nevertheless, travellers from the southern hemisphere leaving during its influenza season could import the virus and transmit it to northern hemisphere attendees who have not yet been vaccinated. To prevent mumps, rubella and influenza, advice should include ensuring up-to-date (or for influenza early) vaccinations, practising proper hygiene and recognizing and reporting signs and symptoms of these diseases.

Although case numbers have been low, measles and IMD outbreaks with international transmission suggest these diseases should also be considered during pre-travel consultations. The endemic measles strain (D5) was last detected in Japan in 2010, and WHO verified elimination in 2015. (*15*) In 2016, however, measles outbreaks occurred in Japan. All were linked to importation, including an outbreak at an international airport. Most cases were undervaccinated. (*16*) For IMD, authors have noted Japan’s low incidence compared to other developed countries. (*17*) A 2015 outbreak with six IMD cases was detected after an international youth event in Japan with more than 33 000 participants from 162 countries. All cases were from Europe, one of which did not attend the event. (*18*) These events show how importation can cause outbreaks even when domestic incidence is low; pre-travel advice should include ensuring up-to-date vaccinations, frequent handwashing and avoiding contact with items that contain others’ saliva or respiratory droplets as much as possible.

Travel advisers should also consider individual traveller behaviours and itineraries. Hepatitis A transmission in Japan has primarily been linked to food, particularly shellfish and seafood. (*19*) This information was obtained through self-reporting, which can be biased by social desirability. In 2017, outbreaks of hepatitis A among men who have sex with men were reported in both Europe and the Americas. (*20*) Individuals who engage in activities that put them at risk for hepatitis A should be advised on preventive measures like vaccination, safe-sex practices, handwashing and food selection. Travellers intending to visit western Japan, especially non-urban areas, should consider JE vaccination and mosquito-bite prevention.

Though not reviewed, rotavirus disease tends to increase from February to May, outside the scheduled MG periods, and tuberculosis has been decreasing since 1999 with 14.4 new cases per 100 000 person-years in 2015. (*21*, *22*)

The selection of diseases for this work was largely based on expert opinion and discussion among leaders within the Infectious Diseases Surveillance Center (IDSC) at NIID. We could have unintentionally left out diseases that might affect travellers visiting the upcoming MGs. Most passive disease surveillance systems may be limited by incomplete reporting, lack of representativeness or failure to identify outbreaks. (*23*) NESID may also suffer these limitations. Additionally, it lacks catchment population data for sentinel surveillance, limiting the ability to estimate sentinel disease incidences. Nonetheless, NESID comprises the most standardized, robust national data available. We believe comparisons across time and place are valid and sufficient for our purposes. In most cases, we attempted to review 10 years of data. For some diseases the introduction or change of reporting requirements prevented that. Readers should conclude with caution when considering diseases with very short reviewed periods.

Few outbreaks associated with sports-based MGs have been reported in literature. Most were reported from the United States of America (*24*-*26*) with one from the United Kingdom of Great Britain and Northern Ireland, (*27*) limiting generalization. Their findings nevertheless imply important considerations: outbreak risk at sports-based MGs is low but not null; outbreaks occur among athletes and nonathletes, associated and unassociated persons and populations of high and low vaccination coverage; importation can spark an outbreak even in low-incidence countries; and, as noted in one article, (*25*) the difficulties of conducting surveillance on international visitors could mean misunderstanding the size or nature of an outbreak or missing an outbreak entirely. Ministries of health, organizations, health-care providers and travellers should ensure up-to-date vaccinations of travellers before they attend MGs, and they should also promote and support travellers carrying updated vaccination records to assist the home country with any potential case or outbreak investigations.

As we have outlined, up-to-date vaccinations with additional preventive measures should be included in pre-travel advice for visitors to the 2019 Rugby World Cup and 2020 Tokyo Summer Olympic and Paralympic Games, specifically for mumps, measles, rubella, influenza and IMD for all travellers and for hepatitis A and JE for travellers at higher risk. When providing advice, health professionals should also inform travellers about the role they could play in transmitting or preventing the transmission of disease to MG attendees from across the world.
